# Erfolgreiche Checkpointinhibition plus extrakorporale Photopherese bei einem Patienten mit metastasiertem Melanom und fortgeschrittenem Sézary-Syndrom

**DOI:** 10.1007/s00105-026-05706-1

**Published:** 2026-05-11

**Authors:** Franziska Jochims, Mirjana Ziemer

**Affiliations:** Department of Dermatology, Venerology und Allergology, University Medical Center Leipzig, Philipp-Rosenthal-Str. 23, 04103 Leipzig, Deutschland

**Keywords:** Sézary-Syndrom, Immuncheckpointinhibitoren, Melanom, Hyperprogression, Remission, Sézary syndrome, Immuncheckpoint inhibitors, Melanoma, Hyperprogression, Remission

## Abstract

Unser Fall zeigt die wirksame kombinierte Therapie mit Ipilimumab und Nivolumab beim SS, die insbesondere in Kombination mit ECP und im Kontext der metastasierten Melanomerkrankung zu einer stabilen partiellen Remission geführt hat. Zu dem befürchteten Hyperprogress ist es nicht gekommen. Der initiale Flare konnte durch eine Therapie mit topischen Glukokortikosteroiden und kurzzeitiges Zuwarten nach dem ersten Zyklus Ipilimumab und Nivolumab gut beherrscht werden. Im längeren Verlauf entwickelte der Patient ein immuntherapiebedingtes lichenoides Arzneimittelexanthem, welches letztlich zu einer Pausierung der Therapie führte. In Folge kam es zum erneuten zerebralen Progress des Melanoms.

## Abstract (en)

Our case clearly demonstrates the effectiveness of combined therapy with ipilimumab and nivolumab in Sézary syndrome, which, particularly in combination with extracorporeal photopheresis (ECP) and in the context of metastatic melanoma, led to stable partial remission. The feared hyperprogression did not occur. The initial flare was successfully bridged by therapy with topical glucocorticosteroids and a short wait after the first cycle of ipilimumab and nivolumab. Over the longer term, the patient developed an immunotherapy-related lichenoid drug eruption, which ultimately led to a pause in therapy and, thus, possibly to renewed cerebral progression of the melanoma. Further clinical research on the mechanism of action of immune checkpoint inhibitors (ICI) in cutaneous T‑cell lymphoma (CTCL) is needed in order to be able to determine the appropriate patients for ICI before starting therapy. ECP could be a possible combination partner in patients with advanced CTCL.

## Anamnese

**Abb. 1:**
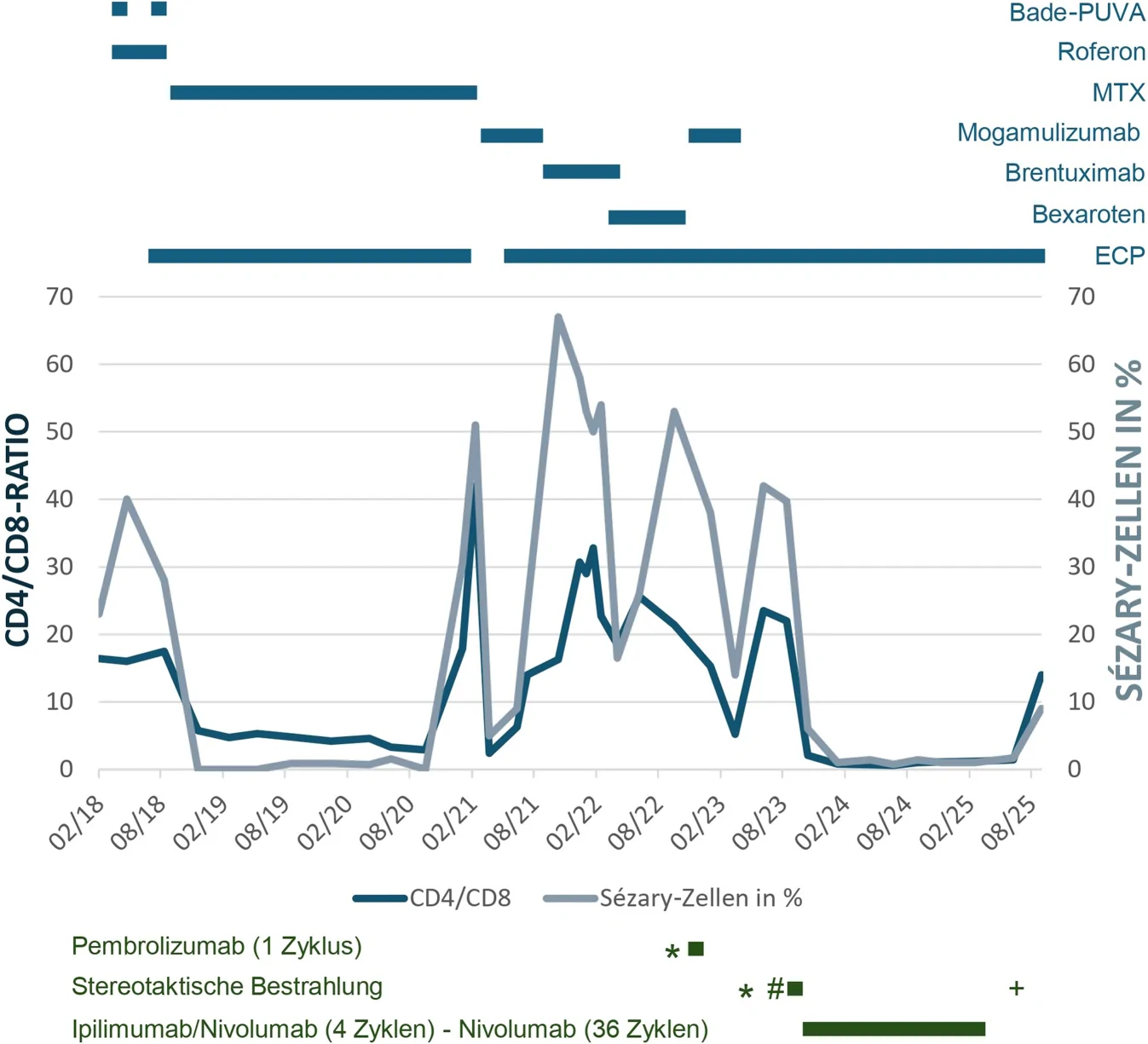
Übersicht der Therapien des Sézary-Syndroms und der 11/2022 neu diagnostizierten Metastasen eines Melanoms bei unbekanntem Primarius. Verlauf der durchflusszytometrischen Parameter CD4:CD8-Ratio und Anteil von Sézary-Zellen im peripheren Blut als Parameter der Krankheitsaktivität des Sézary-Syndroms im zeitlichen Verlauf unter den unterschiedlichen Therapien. * Exzision einer kutanen Melanommetastase, # Nachweis von 8 zerebralen Metastasen, + zerebraler Progress

Wir berichten über einen 84-jährigen Patienten, der sich 2018 erstmalig an unserem Hauttumorzentrum vorstellte. Zum Zeitpunkt der Erstdiagnose bestand ein Sézary-Syndrom (SS) im Stadium IIIB mit Erythrodermie, peripherer Lymphadenopathie, erhöhter CD4/CD8-Ratio (16,4) und durchflusszytometrisch hohem Anteil von Sézary-Zellen (23 %, absolut 2162/µl, CD4+CD7-CD26-) im peripheren Blut. Zwischen 2018 und 2023 wurde der Patient mittels extrakorporaler Photopherese (ECP) in Kombination mit topischen Steroiden und sequenziell mit Bade-PUVA, Interferon-α-2a, Methotrexat, Mogamulizumab, Brentuximab, Bexaroten behandelt, die jeweils kurzzeitig zu partiellen Remissionen führten (Abb. 1).

Im November 2022 wurden an der linken Wange subkutane Knoten getastet, die histologisch als kutan-subkutane Melanommetastase gesichert wurden. Umfangreiche bildgebende und körperliche Untersuchungen erbrachten keinen Anhalt für einen Primärtumor, sodass von einem „melanoma of unknown primary“ ausgegangen werden musste. Die Melanommetastase und ein lokales Rezidiv wurden operativ vollständig entfernt. Nach bereits verabreichter erster Infusion von Pembrolizumab 200 mg (bei BRAF-Wildtyp) wurde die adjuvante Therapie jedoch nach Besprechung im „interdisziplinären multizentrischen Tumorboard Kutane Lymphome“ aufgrund unzureichender Daten und der Befürchtung eines Hyperprogresses des SS nicht fortgesetzt. Im Anschluss an die 1‑malige Gabe von Pembrolizumab kam es kurzfristig zu einem Abfall der CD4/CD8-Ratio und der Sézary-Zellen, der Patient war jedoch im Hinblick auf Erythrodermie, Allgemeinzustandsminderung und Pruritus weiter deutlich symptomatisch.

## Klinischer Verlauf

**Abb. 2:**
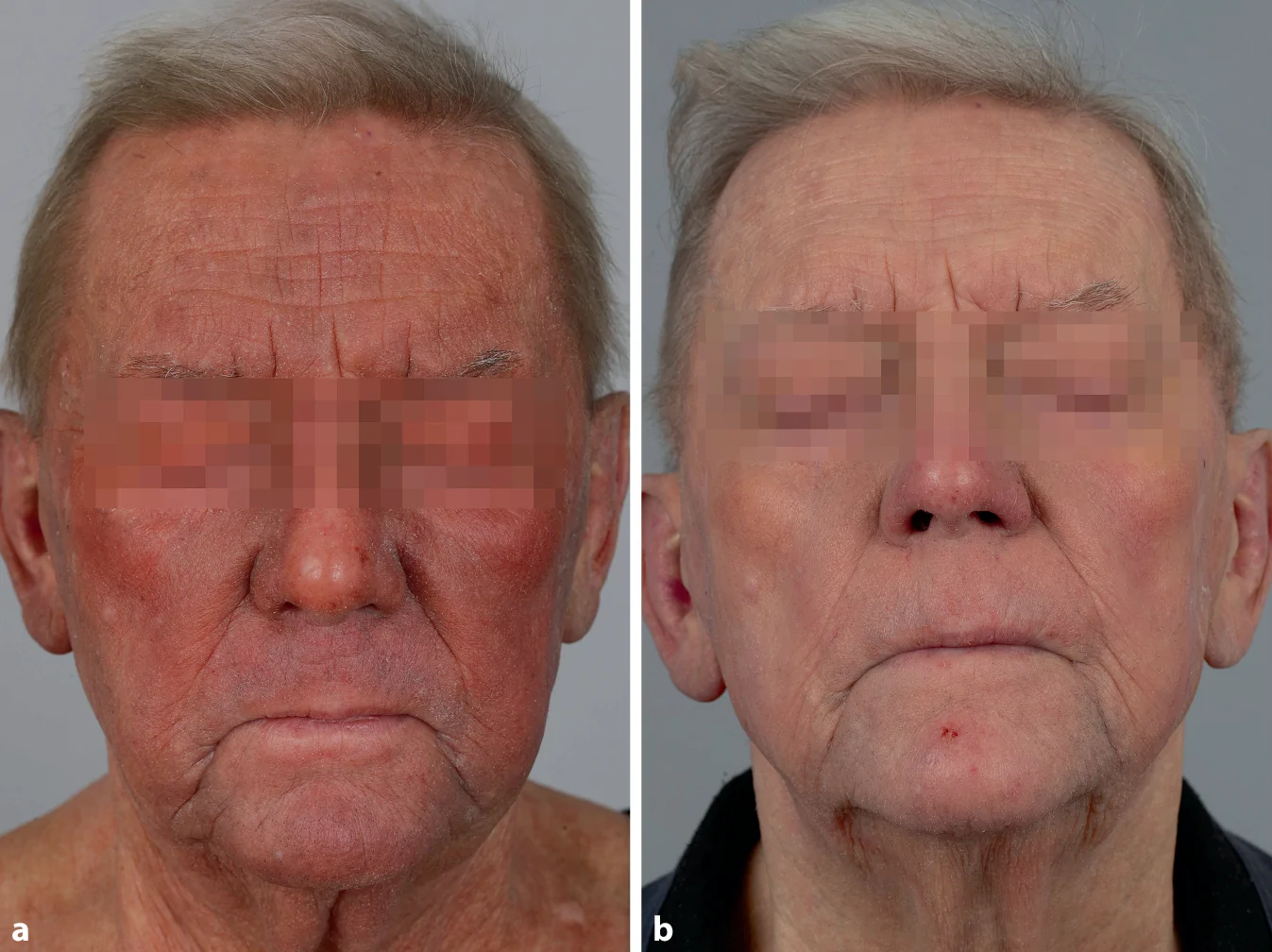
Gesichtsmanifestation des Sézary-Syndroms vor und nach Einleitung der Immuncheckpointinhibition: **a** 06/2023 unter Mogamulizumab und ECP vor Induktion der Immuncheckpointinhibition, **b** 11/2024 Hautbefund nach einem Jahr Immuncheckpointinhibition in Kombination mit der ECP

Im August 2023 erlitt der Patient ein Rezidiv seines Melanoms mit 8 zerebralen Metastasen maximal 4 mm durchmessend. Auch seitens des SS lag ein Progress mit progredienter Lymphknotenschwellung, zunehmender Erythrodermie und steigender CD4/CD8-Ratio (23,5) vor (Abb. 2a und 3a).

**Abb. 3:**
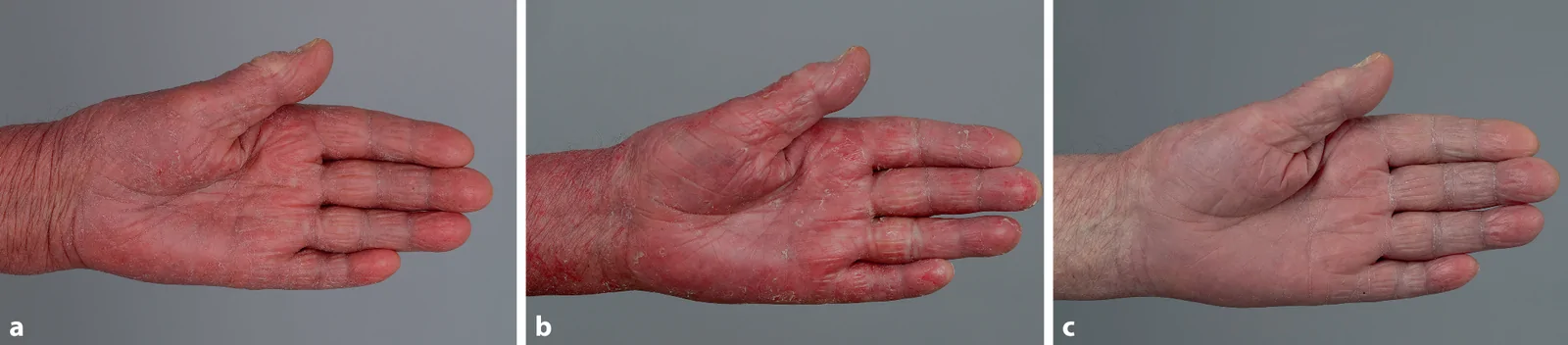
Flare in der Induktionsphase von Ipilimumab/Nivolumab am Beispiel der Handflächen: **a** 06/2023 unter Mogamulizumab und ECP vor Induktion der Immuncheckpointinhibition, **b** 10/2023 Flare nach Induktion mit Ipilimumab/Nivolumab und ECP, **c** 11/2024 Hautbefund nach einem Jahr Immuncheckpointinhibition in Kombination mit ECP

## Diagnose

- Metastasiertes Melanom mit BRAF-Wildtyp bei einem Patienten mit fortgeschrittenem, therapierefraktärem Sézary-Syndrom

## Therapie und Verlauf

Nach Beratung im interdisziplinären Tumorboard wurde Mogamulizumab beendet, und der Patient erhielt nun eine kombinierte Immuncheckpointinhibitor(ICI)-Therapie mit Ipilimumab und Nivolumab (3 mg und entsprechend 1 mg/kgKG), die über insgesamt 4 Zyklen gegeben wurde. Nach dem ersten Zyklus kam es zu einem kurzen klinischen Flare des SS mit progredienter Rötung und Schuppung (Abb. 3b). Die Therapie wurde jedoch nach kurzer Unterbrechung fortgeführt. Innerhalb weniger Wochen kam es zu einer fast kompletten klinischen Remission des SS bei nahezu vollständiger Normalisierung der Blutparameter (11/2023: CD4/CD8-Ratio 2,1; Anteil der Sézary-Zellen im peripheren Blut 6 %, absolut 426/µl) (Abb. 2b und 3c). Die Therapie wurde mit Nivolumab 240 mg alle 2 Wochen fortgeführt. Die zerebralen Metastasen wurden zusätzlich stereotaktisch bestrahlt (Einzel- und Gesamtdosis je 20 Gy). Die ECP wurde alle 2 Wochen beibehalten. Die Therapieverlauf ist in Abb. 1 dargestellt.

**Abb. 4:**
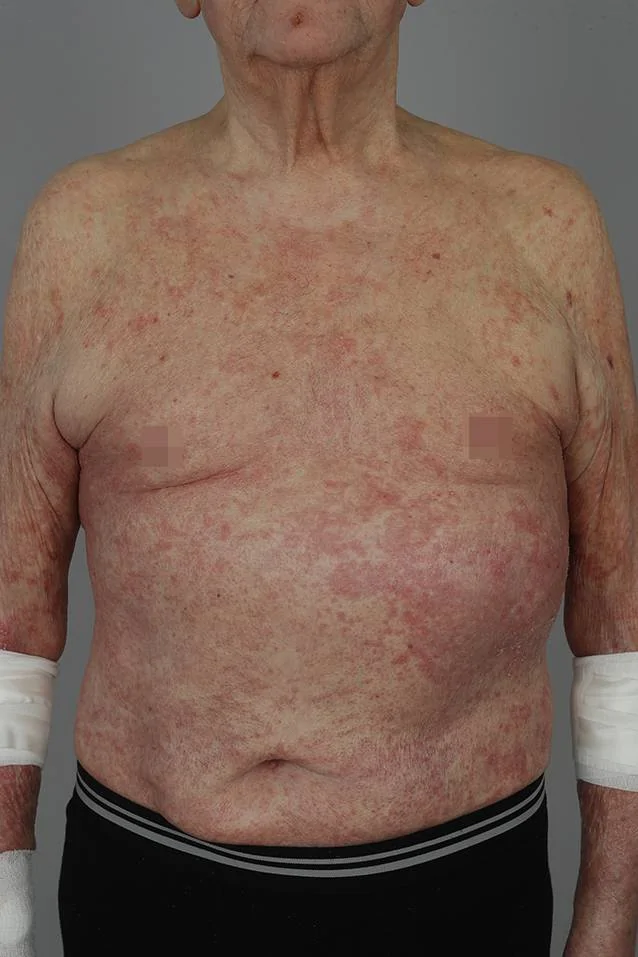
Immuntherapiebedingtes lichenoides Arzneimittelexanthem mit Betonung des Stamms unter Nivolumab-Therapie 07/2025

Der Patient hat unter Fortführung der ECP bis dato 36 Zyklen Nivolumab erhalten. Unter der Therapie mit Nivolumab entwickelte er Anfang 2025 ein immuntherapiebedingtes lichenoides Arzneimittelexanthem (Abb. 4), welches zu größeren Infusionsabständen und letztlich einer 5‑monatigen Pausierung von Nivolumab zwischen April und September 2025 und infolge zu einem erneuten Anstieg der CD4/CD8-Ratio und der Sézary-Zellen im peripheren Blut führte. Im September 2025 erlitt der Patient einen zerebralen Progress mit einer neuen 4 mm messenden Metastase.

Im Oktober 2025 erfolgte die Wiederaufnahme von Nivolumab und im Dezember 2025 die stereotaktische Bestrahlung. Mit der Reinduktion einer Immunkombinationstherapie war man aufgrund des inzwischen reduzierten Allgemeinzustandes des betagten Patienten vorerst zurückhaltend. Letztendlich wurden Ipilimumab und Nivolumab im März 2026 erneut gestartet.

## Diskussion

Leitliniengerechte Therapien beim SS in der Erstlinie umfassen die ECP, Interferon‑α, PUVA und Bexaroten. In der Zweitlinie finden sich die zielgerichteten Therapeutika wie Brentuximab-Vedotin, Mogamulizumab sowie Alemtuzumab, aber auch die Ganzhautelektronenbestrahlung und Chemotherapeutika. Unter den genannten Therapien kommt es bei den meisten Patient:innen jedoch lediglich zu einer partiellen und zeitlich sehr limitierten Remission, sodass zwischen diesen Therapieformen nicht selten im Krankheitsverlauf rotiert werden muss. Insbesondere bei jüngeren Patient:innen kann eine allogene Stammzelltransplantation durchgeführt werden [1]. Allerdings sind auch hier Krankheitsrezidive nicht selten [2].

Vor diesem Hintergrund stellt die Therapie primär kutaner T‑Zell-Lymphome (CTCL) mit ICI einen noch nicht etablierten, aber potenziell vielversprechenden Ansatz dar. Die bislang verfügbaren Daten sind jedoch begrenzt, insbesondere für die CTLA-4-Blockade und Kombinationstherapien, zu denen überwiegend nur Fallberichte vorliegen [3–5]. Die objektive Ansprechrate (ORR) von Patient:innen mit Mycosis fungoides (MF) und SS unter ICI liegt in kleinen Studienkollektiven zwischen 15 % und 48 % [6–9]. Eine Phase-II-Studie mit Pembrolizumab zeigte eine ORR von 38 % [8], während unter Nivolumab geringere Ansprechraten beobachtet wurden [9]. Neuere Daten bestätigen die Heterogenität der Ergebnisse [6, 7]. Dabei scheint eine Monotherapie möglicherweise ebenso effektiv zu sein wie Kombinationstherapien [4, 5].

Die zurückhaltende Anwendung von ICI bei CTCL beruht auf der Besonderheit, dass es sich um maligne T‑Zellen handelt, die selbst Ziel der Immunmodulation sind. Im Gegensatz zu soliden Tumoren besteht die Möglichkeit, dass Tumor-T-Zellen durch Checkpointinhibition aktiviert werden und proliferieren [3, 10].

Dies kann potenziell zu einem Hyperprogress führen. In einer Phase-II-Studie mit Pembrolizumab zeigten jedoch 53 % der Patient:innen lediglich einen transienten Flare, während ein echter Hyperprogress nicht beobachtet wurde [8]. Flare-Reaktionen korrelieren mit einer erhöhten PD-1-Expression auf Tumorzellen und sind initial schwer von einem Progress zu unterscheiden [8]. Während ein Flare ein vorübergehendes inflammatorisches Phänomen darstellt, beschreibt der Hyperprogress eine rasche und anhaltende Krankheitsverschlechterung.

Molekulare Analysen, beispielsweise von PRKCQ oder Komponenten des TCR-Signalwegs, könnten zukünftig helfen, das individuelle Risiko besser abzuschätzen [11]. Insgesamt empfehlen die Autoren, die Therapie initial fortzuführen, da ein Flare unter fortgesetzter ICI-Gabe typischerweise abklingt [8, 9].

Ob die in unserem Fall durchgeführte 2‑wöchentliche ECP einen additiven immunmodulatorischen Effekt auf die ICI ausübt, bleibt bislang spekulativ.

Weder die PD-1-Expression noch die transiente klinische Verschlechterung zeigten eine Assoziation zu einem besseren Therapieansprechen. Dagegen könnten strukturelle Varianten von PD-L1, eine niedrige KIR3DL2-Expression sowie die Expansion von CD8-positiven Effektor-T-Zellen prädiktiv für ein Ansprechen sein und stellen einen weiteren Forschungsansatz zur Auswahl geeigneter CTCL-Patient:innen für eine ICI dar [8, 12, 13].

Als Limitation sehen wir, dass die Evidenz zur Anwendung von ICI bei CTCL überwiegend auf kleinen, heterogenen Patientenkollektiven sowie auf frühen klinischen Studien oder Fallserien basiert [6–9]. Insbesondere für das Sézary-Syndrom sind die Daten sehr limitiert und teilweise widersprüchlich.

## Fazit für die Praxis

ICI stellen einen vielversprechenden, jedoch noch nicht etablierten Therapieansatz bei CTCL dar. Die Anwendung sollte derzeit vorzugsweise im Rahmen individueller Therapieentscheidungen erfolgen, insbesondere bei therapierefraktären Verläufen. Eine sorgfältige klinische Verlaufskontrolle ist essenziell, um Flare-Reaktionen von einer echten Krankheitsprogression abzugrenzen. Zukünftige Studien sind erforderlich, um prädiktive Biomarker zu identifizieren und die optimale Patientenselektion zu verbessern.
